# Supplementary material for: Antigen-specificity of clonally-enriched CD8+ T cells in multiple sclerosis
Source: bioRxiv. 2024 Sep 7:2024.09.07.611010. Preprint. [Version 1] doi: 10.1101/2024.09.07.611010 (PMC11398516; doi:10.1101/2024.09.07.611010)
Supplement: Supplementary file 1 [file media-1.pdf]

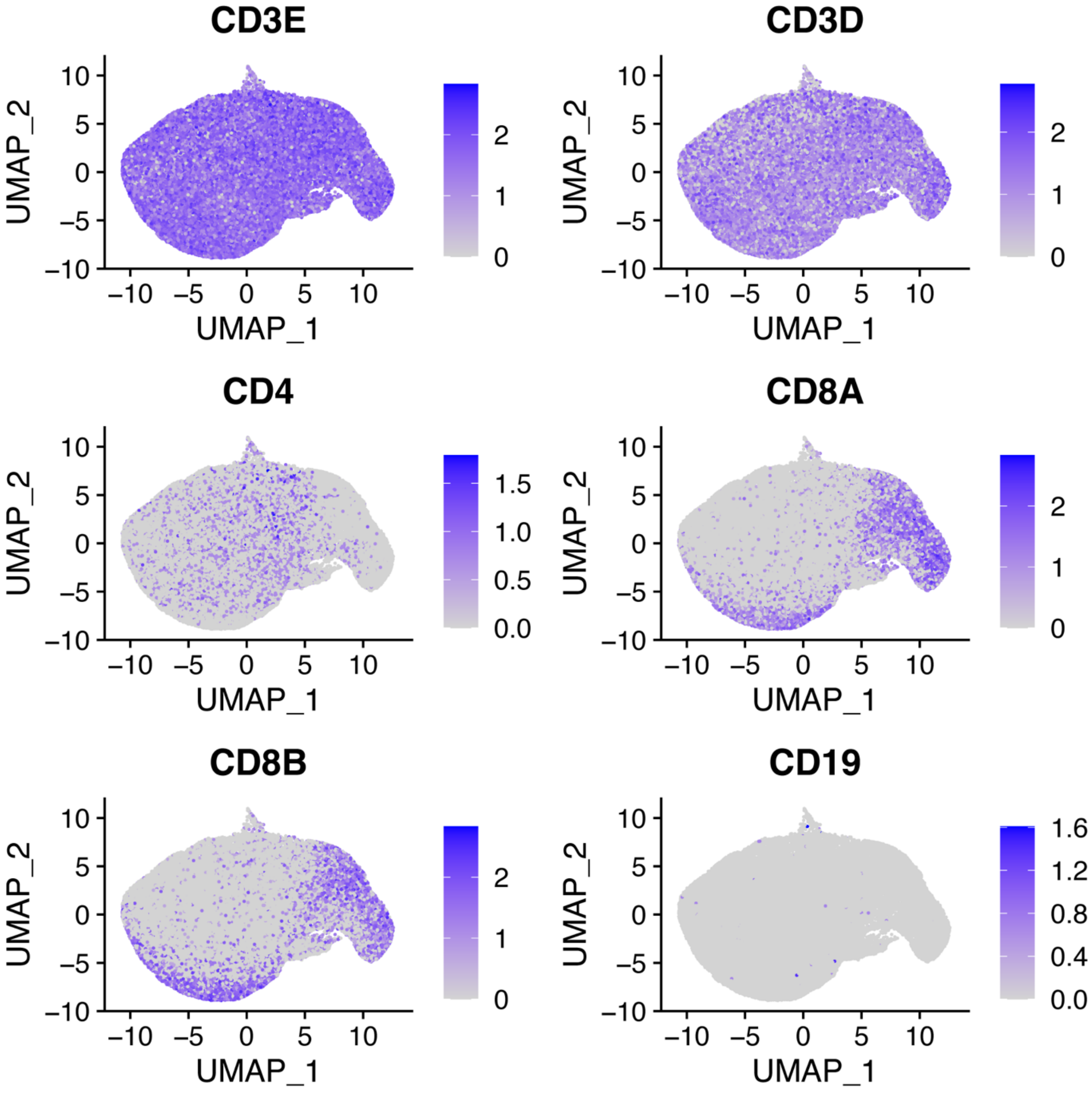

Figure S1. T cell gene expression analysis. Expression for the indicated genes is shown for all T cells (blood and CSF combined) after merging scRNA-seq and scTCR-seq data.

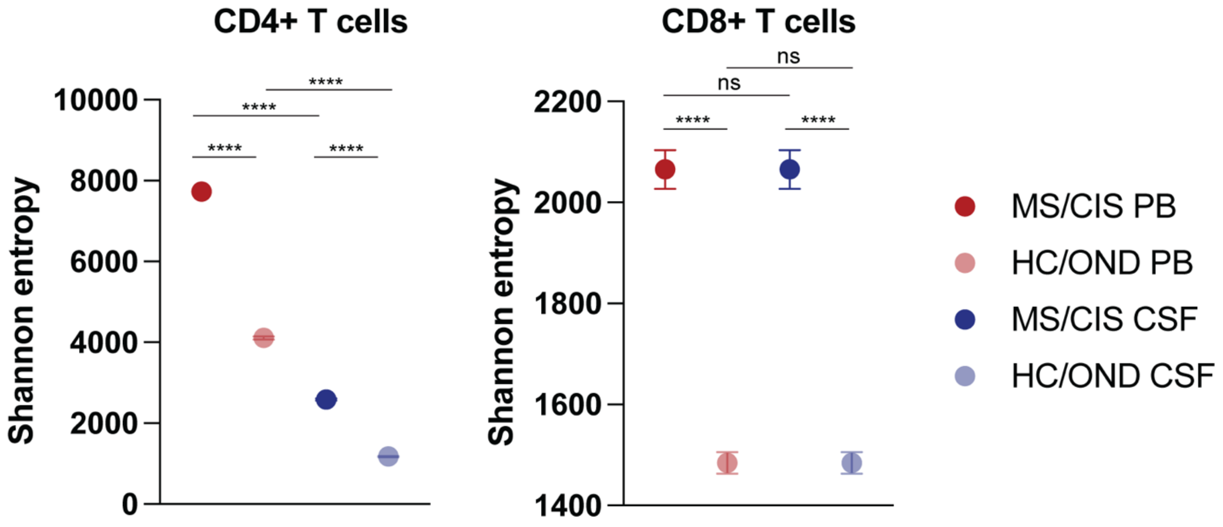

Figure S2. T cell diversity analysis. Shannon entropy analysis (where y-axis indicates exponential of Shannon-Wiener index) is shown for CD8+ T cells in the peripheral blood (PB) and CSF by disease status. Abbreviations: MS = multiple sclerosis; CIS = clinically isolated syndrome; HC = healthy control; OND = other neuroinflammatory disease. ****p < 0.0001.

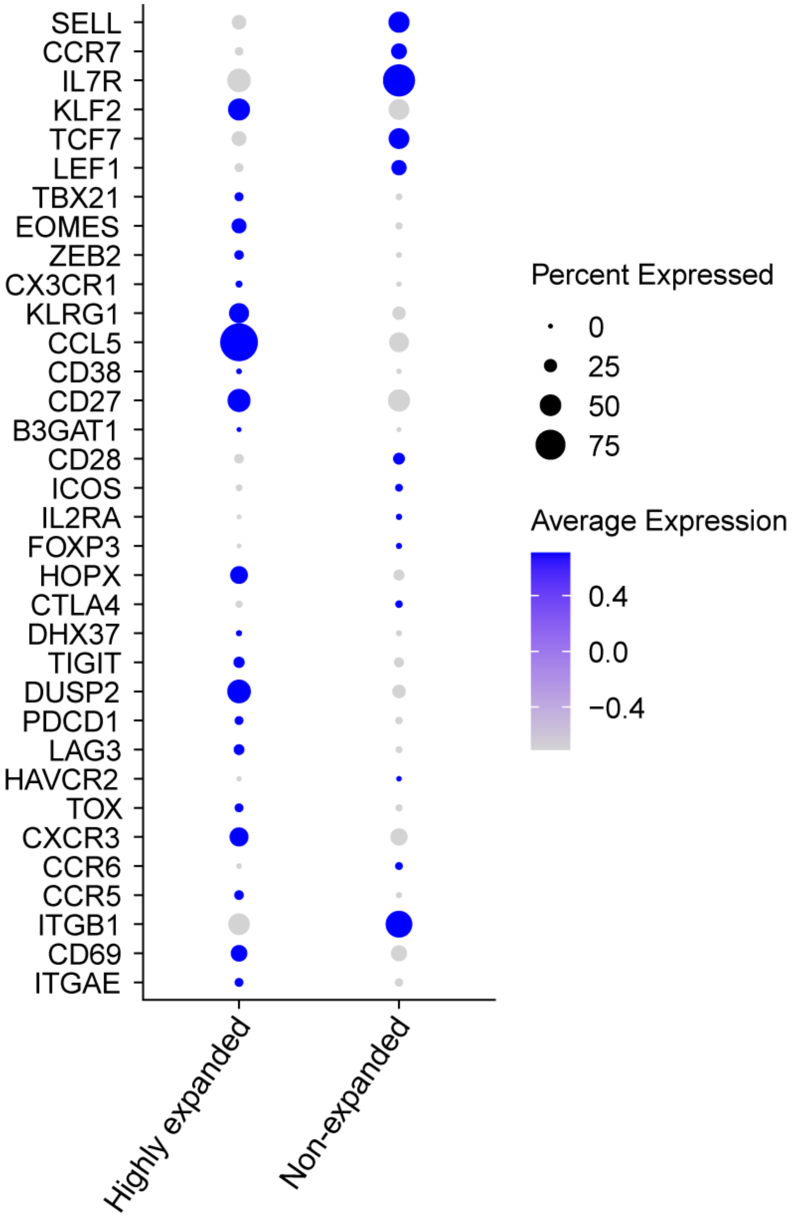

Figure S3. Gene expression analysis of CSF T cells by expansion status. Expression levels and percent expression of the indicated genes is shown for highly expanded CSF T cells versus non-expanded T cell clonoytypes.

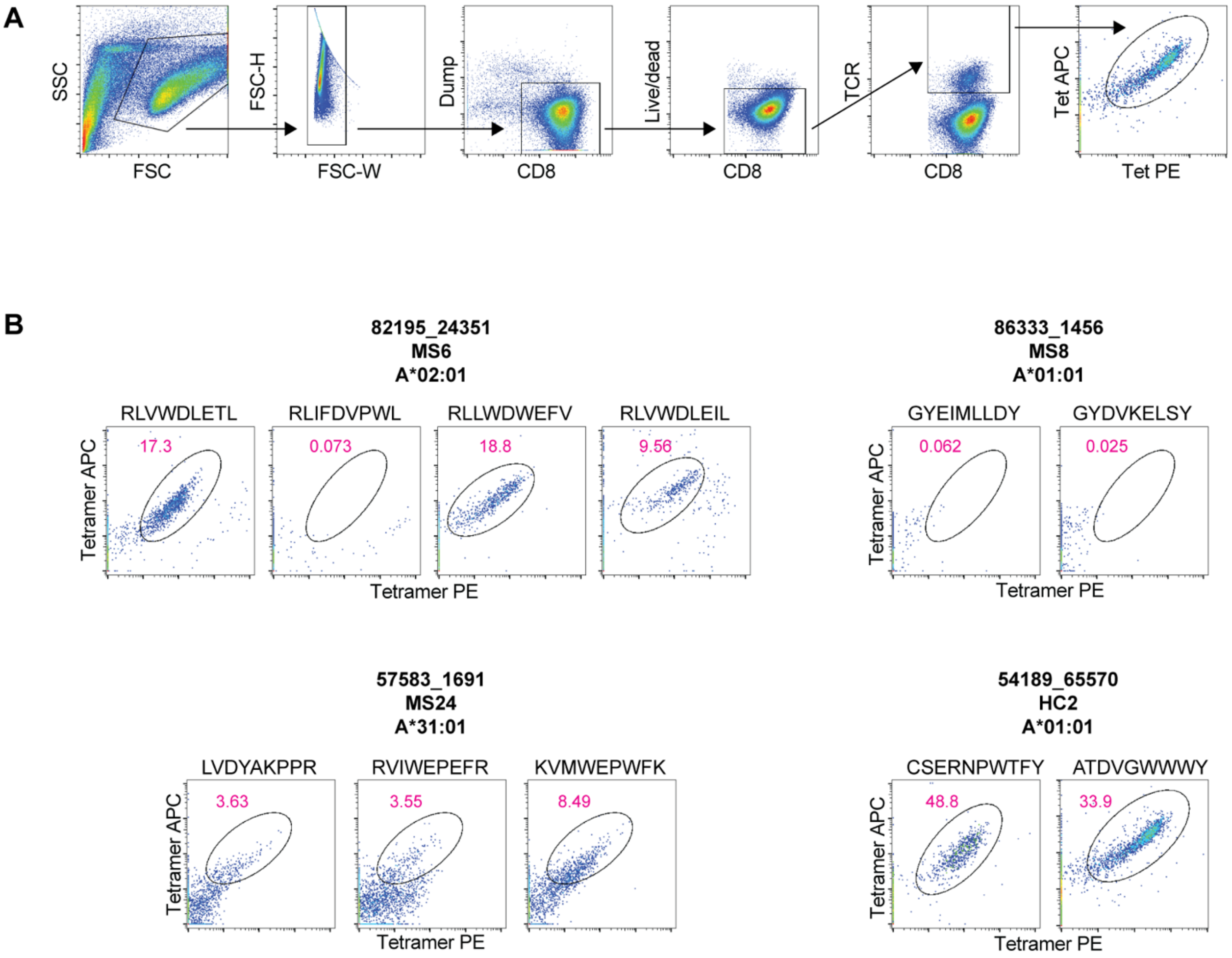

Figure S4. Tetramer screening of antigens identified by pMHC yeast display. Representative flow cytometry analysis of pMHC tetramer stained CD8+ T cells following TCR knockin (A). Four patient-derived TCRs were tested for tetramer binding to the indicated peptides (B).

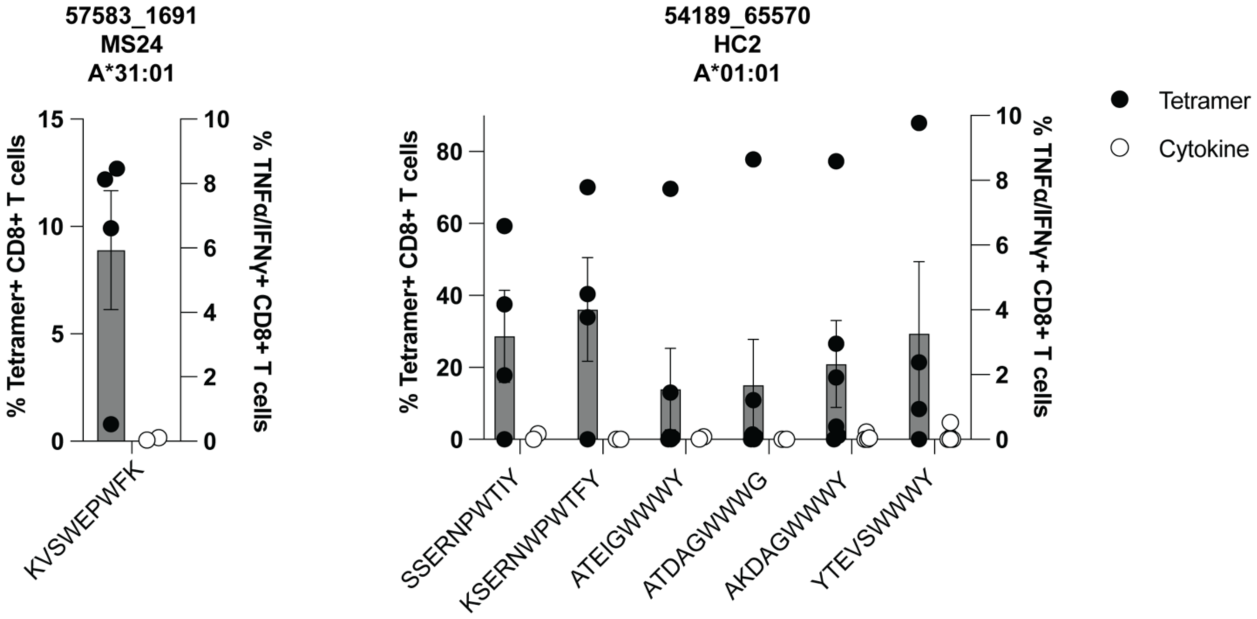

Figure S5. Validation results of peptide homologs to yeast display mimotopes. Summary of tetramer binding and cytokine reactivity of the indicated peptides to two different TCRs. Cytokine reactivity reflects subtracted background from no stimulation control.

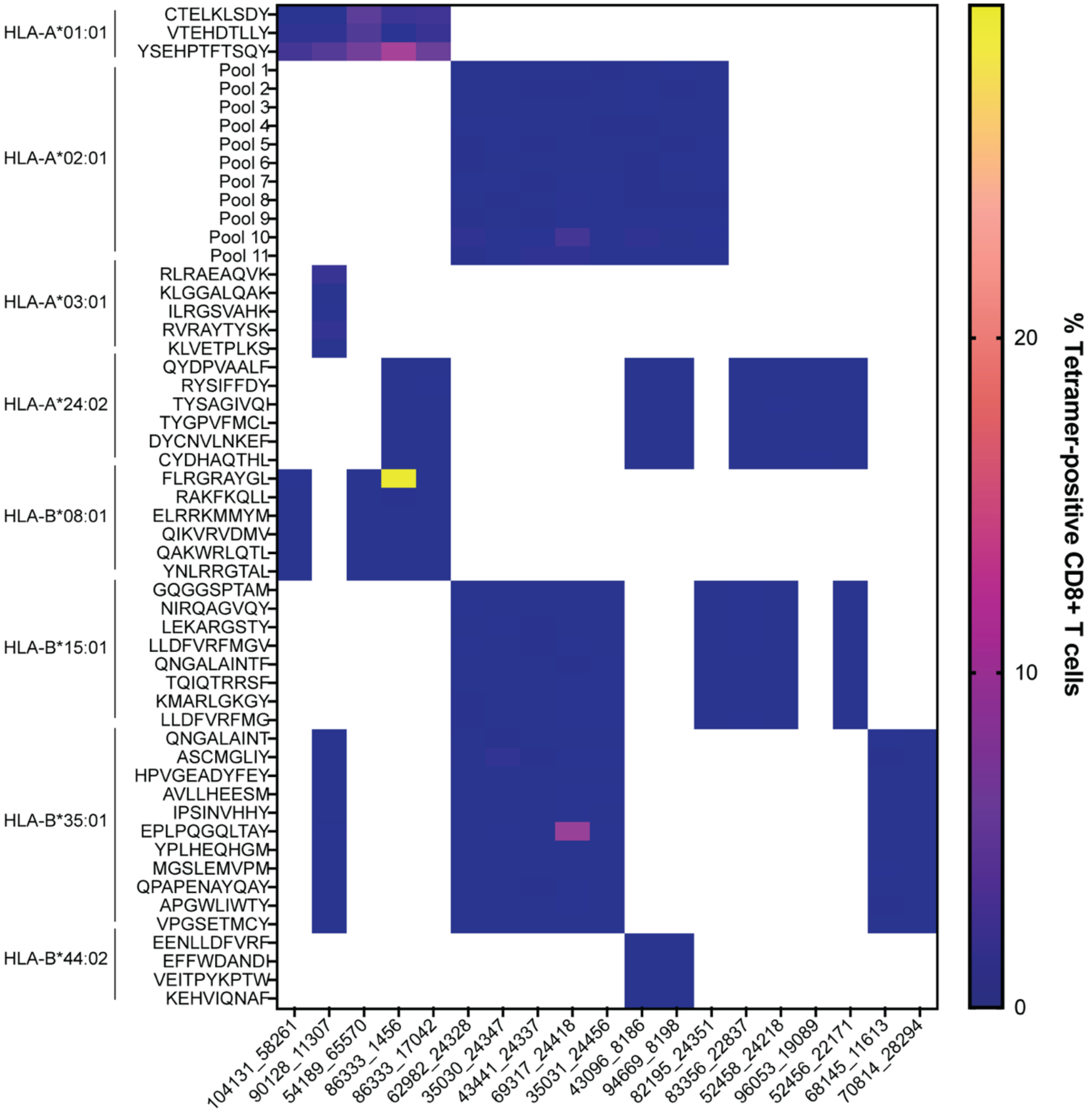

Figure S6. Summary of viral antigen specificity of highly expanded CSF-enriched CD8+ T cells. The summary of all pMHC tetramer screening for 98 viral peptides for 19 patient-derived TCRs is shown in the heatmap. All viral peptides were tested individually except in the case of the peptides for HLA-A*02:01 where tetramers were tested in pools of 5. Each peptide/pool was tested a minimum of two times in using different T cells from different donors.
